# Supplementary material for: Mefloquine reduces the bacterial membrane fluidity of Acinetobacter baumannii and distorts the bacterial membrane when combined with polymyxin B
Source: mBio. 2025 Feb 25;16(4):e04016-24. doi: 10.1128/mbio.04016-24 (PMC11980597; doi:10.1128/mbio.04016-24)
Supplement: Supplemental material — Figure S1 and Table S1. [file mbio.04016-24-s0001.pdf]

Supplementary Figure 1. Mutational Frequency of MFQ against *A. baumannii*. 100-days continuous passage of *A. baumannii* into MHB in the presence of MFQ or rifampin show *A. baumannii* failed to emerge resistance against MFQ.

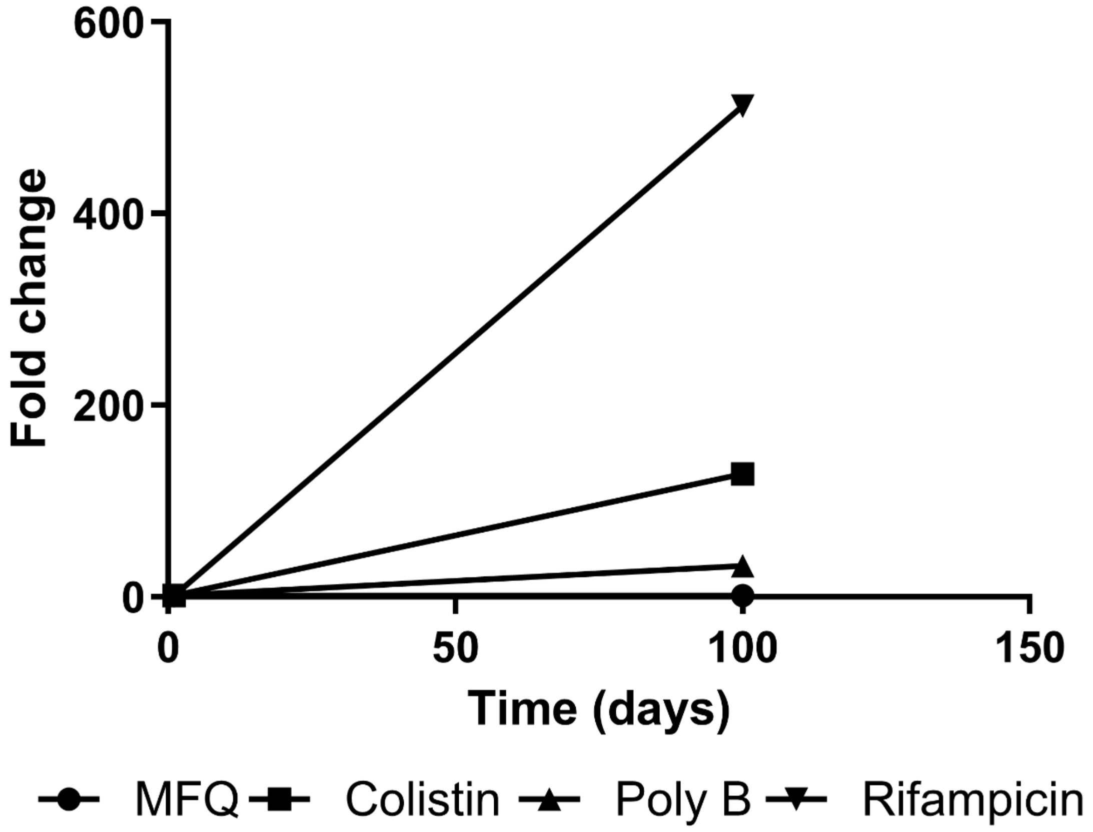

Supplementray Table 1. Details on the MDS models: Number of component molecules, starting distance of the focused compound from the center of the PE/PG membrane bilayer (z = 0 nm), the simulation box dimensions for each system.

**Model no**	**Name**	**Number of molecules PE:PG:CL:MLCL**	**Starting distance of the drug (Å)**	**Box size (Å X Å X Å)**	**Time total (ns)**
I	Drug-free	128:120:14:11	-	65.1X62.7X100.5	1100
II	1Benzene/BL	120:112:12:10	7.1	65.1X62.7X100.5	1012
III	1MFQ/BL	120:112:12:10	7.3	65.1X62.7X100.5	1022
IV	2MFQ/BL	118:101:12:9	6.9	65.1X62.7X100.5	1043
V	3MFQ/BL	109:98:12:9	7.2	65.1X62.7X100.5	1008
VI	4MFQ/BL	90:95:12:9	7.1	65.1X62.7X100.5	968
